# Sex-related differences in clinical outcomes and predictive factors in the very elderly patients with ACS undergoing PCI

**DOI:** 10.3389/fcvm.2022.950165

**Published:** 2022-09-28

**Authors:** Jia-li Wang, Xiao-quan He, Chun-yan Guo, Hui Chen, Hong-wei Li, Shu-mei Zhao

**Affiliations:** ^1^Department of Cardiology, Cardiovascular Center, Beijing Friendship Hospital, Capital Medical University, Beijing, China; ^2^Department of Internal Medical, Medical Health Center, Beijing Friendship Hospital, Capital Medical University, Beijing, China; ^3^Beijing Key Laboratory of Metabolic Disorder Related Cardiovascular Disease, Beijing, China

**Keywords:** very elderly patients, acute coronary syndromes, percutaneous coronary intervention, sex differences, major adverse cardiovascular and cerebrovascular events

## Abstract

**Background:**

As shown in previous studies, there may be sex-related differences in clinical outcomes in patients with acute coronary syndromes (ACS) after percutaneous coronary intervention (PCI). However, the benefits of PCI in very elderly ACS patients and the gender differences were poorly described and understood. We investigated the clinical characteristics and outcomes after PCI by sex stratification, and the predictive factors of major adverse cardiovascular and cerebrovascular events (MACCE) in this very elderly ACS cohort.

**Methods:**

A total of 1,676 consecutive ACS patients (50.2% women) aged ≥80 years old between January 2013 and May 2020 were recruited in this study. All patients were divided into four groups according to gender and treatment: male PCI (*n* = 321) and conservative management groups (*n* = 513), and female PCI (*n* = 283) and conservative management groups (*n* = 559). Clinical and coronary lesion characteristics were compared among four groups, also the clinical outcomes. MACCE and their predictive factors were assessed using Kaplan–Meier curve and Cox regression analysis.

**Results:**

PCI procedures were conducted in 604 patients, and 1,072 were conservative management. Men were most likely to present with prior myocardial infarction (MI), peripheral artery disease, and chronic total occlusion (CTO); women had a higher prevalence of hypertension and dyslipidemia. The proportion of men receiving PCI procedures was significantly higher than that of women (38.5 vs. 33.6%, *p* = 0.038). Compared to conservative management, successful PCI significantly improved composite MACCE in both men (33.9 vs. 18.4%, *p* < 0.001) and women (27.9 vs. 20.8%, *p* = 0.026). There were no differences between sex in the improvement of clinical outcomes after PCI. In addition, age, ST-segment elevation myocardial infarction (STEMI), log N-terminal pro-brain natriuretic peptide (NT-proBNP), P2Y12 receptor antagonist, and β-blocker were independently associated with the incidence of MACCE after PCI tested by the Cox regression model, but not gender (male: hazard ratio (HR) 1.275, 95% confidence interval (CI) 0.853–1.905, *p* = 0.236).

**Conclusion:**

In this very elderly ACS cohort, men presented with more complex clinical conditions, and women were less likely to receive PCI treatment. Both women and men had similar benefits from the PCI procedure in the decrease of MACCE.

## Background

Acute coronary syndrome (ACS) has been the leading cause of death in the past few decades. Fortunately, the application of percutaneous coronary intervention (PCI) had significantly improved the clinical outcomes in these patients (1, 2). With the progress of aging worldwide, very elderly patients with ACS have formed a large population, who need timely and effective treatment. However, this population had received significantly less invasive angiography and PCI treatment in clinical practice (3, 4), and was often excluded from large multicenter clinical studies (5, 6), because of possible higher risk of complications and mortality (7, 8), as well as the cardiologists may have fewer experiences and knowledge of PCI treatment in very elderly ACS patients.

Previous studies have also revealed that there are sex-related differences in clinical characteristics, outcomes, and quality of life (QoL) in patients with ACS after PCI. For example, women are more likely to have atypical symptoms and nonobstructive coronary disease on angiography (9–11), and less likely to receive guideline-based therapies or cardiac rehabilitation (9, 12). Women often have higher rates of peri-procedural complications and mortality with PCI (12–14). Independent of the ACS presentation or comorbidities, the female sex was a predictor of poorer QoL following PCI for ACS (15), including significantly higher pain, anxiety, and depression. However, there are few specific descriptions of sex-related influence on the very elderly ACS patients after PCI. Awareness of these differences in the very elderly population may lead to improved sex-based diagnosis and treatment strategies, as well as the assessment of prognosis.

The aims of the present study were to investigate the sex-related differences in clinical characteristics, effectiveness, and safety of PCI treatment, and predictive factors of major adverse cardiovascular and cerebrovascular events (MACCE) after PCI in very elderly ACS patients.

## Materials and methods

### Study population and protocol

**Figure 1:**
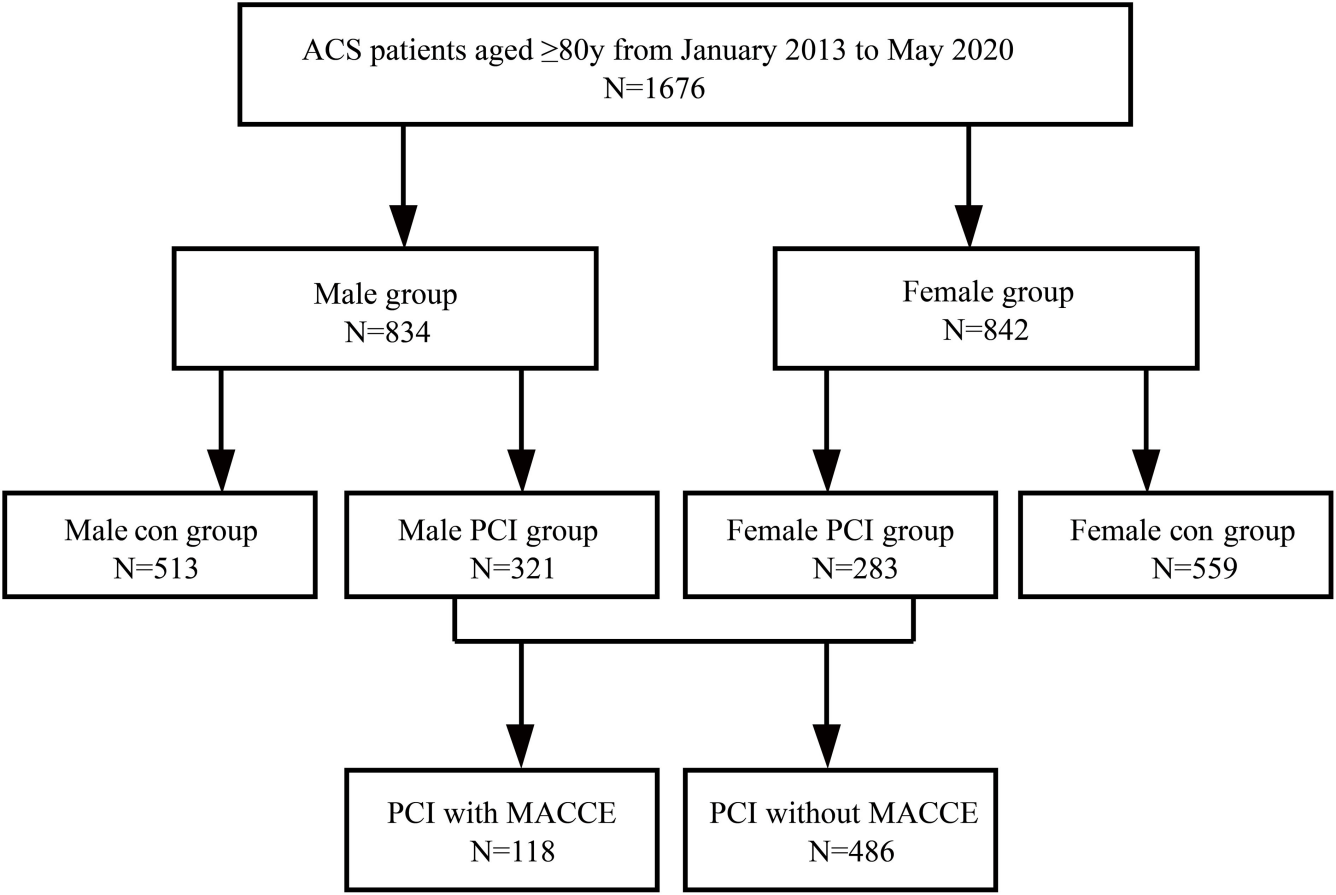
Patients' enrollment flow. ACS, acute coronary syndrome; PCI, percutaneous coronary intervention; MACCE, major adverse cardiovascular and cerebrovascular events; con, conservative management.

We retrospectively enrolled ACS patients aged 80 years or older (*n* = 1,676) between January 2013 and May 2020, who entered the Cardiovascular Center of Beijing Friendship Hospital Database (CBD) Bank. The study flow chart and protocol were described in Figure 1: (1) Consecutive ACS patients aged ≥ 80 years old (*n* = 1,676) were enrolled. (2) All the patients were classified by gender: female group (*n* = 842), and male group (*n* = 834). (3) Within the male group, patients were categorized into the male PCI group (M-PCI group, *n* = 321) and the male conservative management group (M-con group, *n* = 513). (4) Within the female group, patients were divided into the female PCI group (F-PCI group, *n* = 283) and the female conservative management group (F-con group, *n* = 559). (5) All the patients undergoing PCI were divided into groups with (*n* = 118) and without MACCE (*n* = 486), according to whether MACCE occurred or not during the follow-up period. Clinical characteristics on admission and incidences of MACCE during follow-up were compared in different sex groups and treatment groups. Meanwhile, predictive factors for MACCE after PCI were detected and assessed in this very elderly cohort.

ACS, including ST-elevation myocardial infarction (STEMI), non-ST-elevation MI (NSTEMI), and unstable angina pectoris (UAP), were diagnosed by symptoms, electrocardiogram (ECG) changes, and cardiac biomarkers. The treatment options (conservative management or PCI) were decided by two cardiologists simultaneously based on international standards and guidelines (16). The study was approved by the Institutional Ethics Committee of Beijing Friendship Hospital and was also in accordance with the 1964 Declaration of Helsinki and its later amendments or comparable ethical standards.

### Clinical outcomes and covariates

The patients' demographic information, initial clinical presentation at admission, and past medical history (hypertension, diabetes, dyslipidemia, prior MI/stroke, and smoking) were retrospectively collected from the clinical information database. Laboratory examination results included high sensitivity C-reactive protein (hs-CRP), N-terminal pro-brain natriuretic peptide (NT-proBNP), creatine kinase-MB (CK-MB), hemoglobin A1c (HbA1C), lipid spectrum, and creatinine that were measured during hospitalization. M-mode and two-dimensional echocardiography (ECHO) were performed (Philips IE33 or EPIQ 7C) for routine parameters, such as left ventricular end-diastolic diameter (LVEDD) and ejection fraction (LVEF). The characteristics of stent implantation and coronary artery were detected by angiographic and PCI procedures, presented in the medical records.

Chronic total occlusion (CTO) referred to 100% coronary artery occlusion with thrombolysis in myocardial infarction (TIMI) grade 0 flow and angiographic evidence of occlusion duration > 3 months (17). Successful PCI was defined as final residual diameter stenosis of < 20% by visual estimation and the presence of normal epicardial coronary flow (TIMI-3 flow) (18). Major hemorrhage was defined by TIMI criteria as hemoglobin decrease ≥5 g/dl or any intracranial bleeding (19). Medication after discharge was determined from the medical records or regular telephone follow-up. The primary endpoint was composite MACCE, which was the combination of non-fatal MI, stroke, heart failure requiring hospitalization (HFRH), and cardiovascular (CV) death. The secondary endpoints referred to all-cause death and each of the adverse events mentioned above. All MACCEs were confirmed by two separate cardiologists simultaneously. Regular follow-up was conducted by clinic visits or phone interviews every 1–3 months until May 2021.

### Statistical analysis

Continuous variables were expressed as median with interquartile range and were compared by the Mann–Whitney *U* test. Categorical data were expressed as frequencies or percentages and were compared by Chi-square or Fisher's exact statistics. The events of primary and secondary endpoint were compared among F-PCI, F-con, M-PCI, and M-con groups by Chi-square test. Logistic regression analysis was conducted to identify the possible factors related to PCI treatment decision-making in the very elderly ACS cohort. Hazard ratio (HR) and 95% confidence interval (CI) were estimated by adjusting for potential confounders including sex, age, STEMI, prior stroke, smoking, creatinine, and NT-proBNP for analyses. Survival curves were conducted by the Kaplan–Meier method and compared with the log-rank test. Cox proportional hazards analysis was used to detect and evaluate predictors for the incidence of composite MACCE between the patients with and without MACCE after PCI treatment. The multivariable model was adjusted for the following covariates in an all-enter way: age, sex, BMI, STEMI, NT-proBNP, creatinine, aspirin, P2Y12 receptor antagonist, statin, and β-blocker. A two-sided *p* < 0.05 was statistically significant. All the statistical analysis was conducted by the Statistical Product and Service Solutions (SPSS) software version 23.0 (IBM, Armonk, NY, USA).

## Results

### Sex differences in clinical and coronary artery characteristics

**Figure 2:**
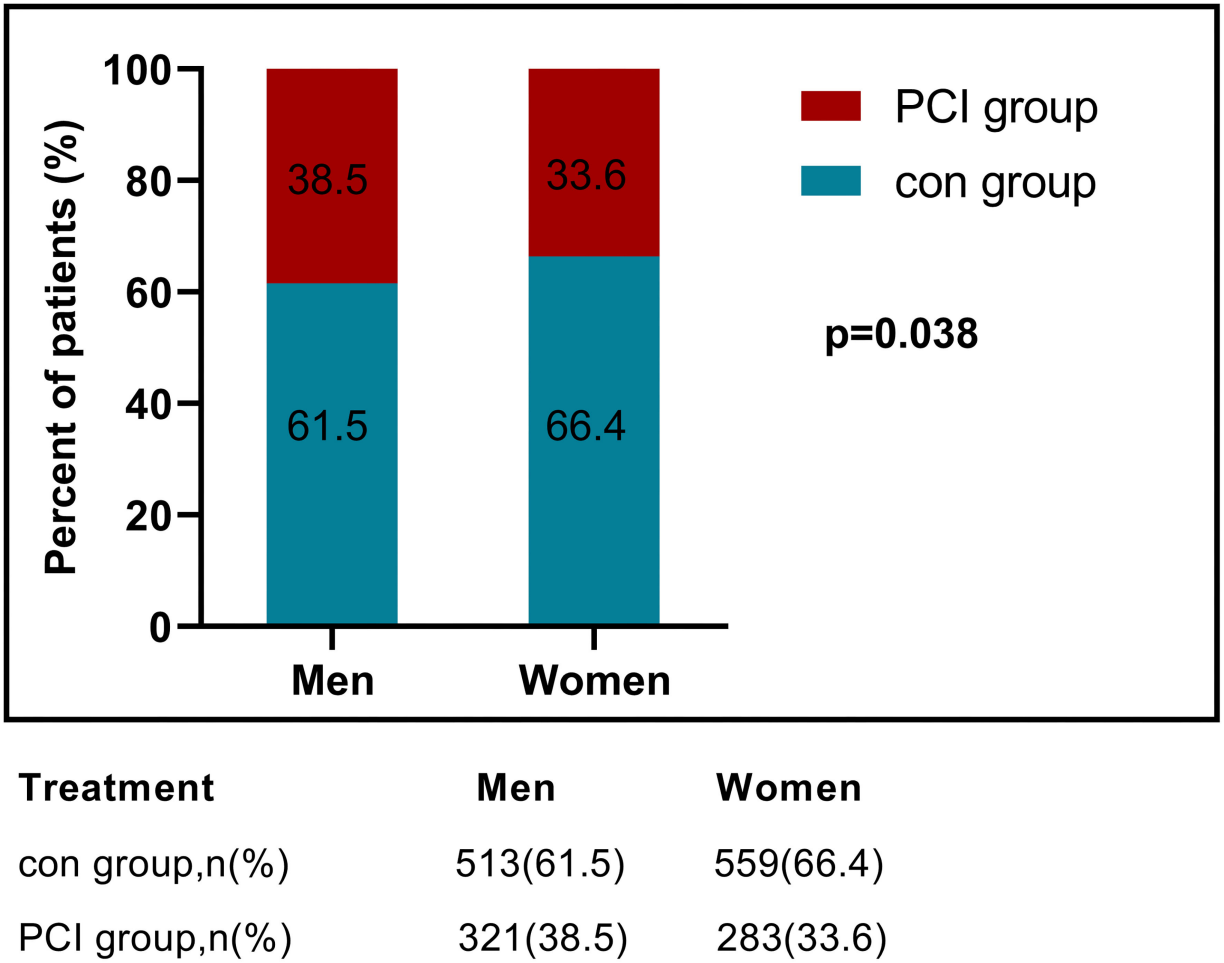
Treatment decisions according to gender. PCI, percutaneous coronary intervention; con, conservative management.

The median follow-up duration of the study was 48 months (interquartile range, 24–60 m). The clinical characteristics of this very elderly ACS cohort were summarized in Table 1. Of the 1,676 patients, there was a similar proportion of men and women. Age, BMI, and types of clinical diagnosis were also matched between the sexes. But the rate of male ACS patients receiving PCI procedures was significantly higher than that of female patients (*p* = 0.038) (Figure 2). In past medical history, male patients had a higher prevalence of prior MI (*p* < 0.001) and peripheral artery disease (PAD, *p* = 0.011), and female patients had a greater history of hypertension (*p* < 0.001) and dyslipidemia (*p* = 0.011). As for the laboratory finding, male patients showed significantly higher levels of hs-CRP, creatinine, CK-MB, and LVEDD, compared to female patients. Meanwhile, female patients were detected to have noticeably higher levels of low-density lipoprotein cholesterol (LDL-C), triglyceride (TG), HbA1C, and proportion of LVEF≥50%. During the follow-up period, the percentage of men taking aspirin was significantly higher than that of women (*p* = 0.005).

**Table 1:** Baseline clinical characteristics in very elderly ACS patients.

**Variables**	**Male ACS**	**Female ACS**	***p* value**	**Male-PCI**	**Female-PCI**	***p* value**
	**(*n =* 834)**	**(*n =* 842)**		**(*n =* 321)**	**(*n =* 283)**	
Age, years	82 (81,85)	82 (81,84)	0.994	82 (81,84)	82 (81,84)	0.887
BMI, kg/m^2^	24.33 (22.09,26.68)	24.44 (22.22,26.94)	0.808	24.22 (22.31,26.46)	24.43 (22.27,26.77)	0.763
**Initial presentation**						
Heart rate, beats/min	70 (62,80)	70 (64,80)	0.227	69 (61,79)	70 (64,80)	0.034
Systolic BP, mmHg	133 (120,147)	133 (121,148)	0.279	132 (121,147)	135 (120,150)	0.151
Diastolic BP, mmHg	71 (64,80)	70 (63,80)	0.024	71 (64,80)	70 (63,80)	0.217
**Past medical history**						
Hypertension, n (%)	603 (72.3)	700 (83.1)	< 0.001	229 (71.3)	227 (80.2)	0.011
Diabetes mellitus, n (%)	273 (32.7)	303 (36.0)	0.161	103 (32.1)	105 (37.1)	0.196
Dyslipidemia, n (%)	302 (36.2)	356 (42.3)	0.011	111 (42.0)	96 (41.4)	0.931
Prior MI, n (%)	129 (15.5)	80 (9.5)	< 0.001	49 (15.3)	24 (8.5)	0.011
Prior stroke, n (%)	226 (27.1)	220 (26.1)	0.653	73 (22.7)	67 (23.7)	0.786
Smoking, n (%)	171 (20.5)	73 (8.7)	< 0.001	75 (23.4)	31 (11.0)	< 0.001
Prior PAD, n (%)	97 (11.6)	67 (8)	0.011	37 (11.5)	23 (8.1)	0.163
**Clinical diagnosis**						
UAP, n (%)	481 (57.7)	513 (60.9)	0.175	133 (41.4)	130 (45.9)	0.265
NSTEMI, n (%)	193 (23.3)	184 (21.9)	0.490	79 (24.6)	65 (23.0)	0.636
STEMI, n (%)	159 (19.1)	145 (17.2)	0.327	109 (34.0)	88 (31.1)	0.454
Length of stay, days	7 (6,10)	7 (6,10)	0.635	8 (6,11)	7 (6,10)	0.088
**Laboratory finding**						
Hs-CRP	3.28 (1.06,14.45)	2.78 (0.94,11.77)	0.042	3.38 (1.37,15.15)	3.44 (1.14,11.28)	0.366
TC, mmol/L	3.74 (3.17,4.38)	4.33 (3.65,5.06)	< 0.001	3.80 (3.25,4.38)	4.45 (3.71,5.15)	< 0.001
LDL-C, mmol/L	2.06 (1.65,2.54)	2.41 (1.89,2.94)	< 0.001	2.13 (1.73,2.54)	2.50 (2.02,3.06)	< 0.001
TG, mmol/L	1.03 (0.76,1.36)	1.26 (0.92,1.72)	< 0.001	1.08 (0.76,1.37)	1.30 (0.94,1.84)	< 0.001
HDL-C, mmol/L	1.02 (0.89,1.19)	1.17 (0.99,1.39)	< 0.001	1.01 (0.87,1.16)	1.14 (0.97,1.37)	< 0.001
Creatinine, umol/L	95.0 (83.5,112.38)	77.7 (65.9,95.5)	< 0.001	91.8 (80.8,104.3)	75.0 (64.0,90.4)	< 0.001
ALT, u/L	15 (11,23)	13 (10,21)	< 0.001	18 (13,30)	15 (10,22)	0.001
HbA1c, %	6.1 (5.6,6.7)	6.1 (5.7,7.0)	0.033	6.1 (5.6,6.9)	6.2 (5.7,7.1)	0.150
First Glu, mmol/L	5.45 (4.73,6.49)	5.38 (4.84,6.74)	0.184	5.58 (4.77,6.56)	5.60 (4.86,7.19)	0.202
CK-MB, ng/ml	1.9 (1.2,4.4)	1.5 (1.0,3.2)	< 0.001	2.75 (1.40,8.93)	1.70 (1.0,4.50)	< 0.001
log NT-proBNP	3.03 (2.52,3.60)	3.0 (2.50,3.56)	0.670	2.96 (2.49,3.44)	2.98 (2.56,3.46)	0.360
LVEF ≥ 50%, n (%)	630 (78.9)	707 (87.5)	< 0.001	247 (78.4)	241 (87.0)	0.006
LVEDD, mm	5.23 (4.90,5.65)	4.90 (4.62,5.23)	< 0.001	5.20 (4.90,5.60)	4.90 (4.64,5.20)	< 0.001
**Medication during follow up**
Aspirin	638 (76.5)	593 (70.4)	0.005	301 (93.8)	237 (83.7)	< 0.001
P2Y12 receptor*	436 (52.3)	438 (52.0)	0.915	290 (90.3)	258 (91.2)	0.728
ACEI/ARB	405 (48.6)	390 (46.3)	0.358	177 (55.1)	145 (51.2)	0.337
β-blocker	482 (57.8)	480 (57.0)	0.745	214 (66.7)	178 (62.9)	0.333
Statin	650 (77.9)	666 (79.1)	0.563	278 (86.6)	247 (87.3)	0.806

^*^P2Y12 receptor antagonist within 12 months after PCI. p, level of statistical significance.

BMI, body mass index; BP, blood pressure; MI, myocardial infarction; PAD, peripheral artery disease; UAP, unstable angina pectoris; NSTEMI, non-ST-segment elevation myocardial infarction; STEMI, ST-elevation myocardial infarction; hs-CRP, high sensitivity C-reactive protein; TC, total cholesterol; LDL-C, low-density lipoprotein cholesterol; TG, triglyceride; HDL-C, high-density lipoprotein cholesterol; ALT, alanine aminotransferase; HbA1c, Hemoglobin A1c; Glu, glucose; CK-MB, creatine kinase-MB; NT-proBNP, N-terminal pro-brain natriuretic peptide; LVEF, left ventricular ejection fraction; LVEDD, left ventricular end-diastolic diameter; ACEI, angiotensin-converting enzyme inhibitors; ARB, angiotensin receptor blockers.

As described in Table 2, the rate of left main (LM) lesions and three-vessel lesions were similar between male and female ACS patients who underwent PCI procedures. However, the rate of CTO in males was higher than that in females (*p* = 0.007). There were no significant differences in post-PCI TIMI 3 flow, procedural success rate, and stent number between the males and females.

**Table 2:** Coronary artery characteristics of patients in the PCI group.

**Variables**	**Male-PCI**	**Female-PCI**	***p* value**
	**(*n* = 321)**	**(*n* = 283)**	
Primary PCI, n (%)	61(19.0)	63(22.3)	0.323
LM disease, n (%)	71 (22.2)	50 (17.7)	0.167
Three-vessel lesion, n (%)	288 (90.0)	241 (85.2)	0.071
CTO rate, n (%)	43 (13.4)	19 (6.7)	0.007
Post-PCI TIMI 3 flow, n (%)	298 (95.8)	273 (96.8)	0.525
Procedural success rate, n (%)	303 (97.1)	273 (96.8)	0.828
Stent number ≥ 2, n (%)	146 (46.8)	114 (40.4)	0.118
IABP use, n (%)	13 (4.0)	12 (4.2)	0.907
Major bleeding, n (%)	10 (3.1)	16 (5.7)	0.125

p, level of statistical significance.

PCI, percutaneous coronary intervention; LM, left main; CTO, chronic total occlusion; TIMI, thrombolysis in myocardial infarction; IABP, intra-aortic balloon pump.

### Clinical factors related to PCI decision making

All the baseline variables entered the logistic regression analysis to detect factors related to PCI decision-making. Based on variables identified from the univariate analysis, a multivariate analysis was conducted to screen out the independent related factors. As detailed in Table 3, the male was more likely to receive PCI treatment, which was 1.34 times higher than that of the female (HR = 1.337, *p* = 0.017). Furthermore, STEMI significantly drove the option of PCI strategy. However, with the declines in age, creatinine, and log NT-proBNP, it was more inclined to the PCI treatment.

**Table 3:** Logistic regression analyses for factors related to PCI decision-making in very elderly ACS patients.

**Variables**	**Univariate regression**			**Multivariate regression**		
	**HR**	**95% CI**	***p* value**	**HR**	**95% CI**	***p* value**
Age	0.937	0.905–0.971	< 0.001	0.925	0.888–0.964	< 0.001
Male	1.236	1.012–1.509	0.038	1.337	1.054–1.697	0.017
STEMI	4.365	3.358–5.675	< 0.001	5.797	4.303–7.809	< 0.001
Prior stroke	0.755	0.60–0.951	0.017	0.808	0.624–1.045	0.104
Smoking	1.441	1.094–1.898	0.009	1.313	0.959–1.797	0.089
Creatinine, umol/L	0.992	0.988–0.995	< 0.001	0.991	0.987–0.994	< 0.001
Hs-CRP	1.008	0.999–1.017	0.083			
log NT-proBNP	0.836	0.722–0.969	0.017	0.803	0.669–0.964	0.019

p, level of statistical significance.

PCI, percutaneous coronary intervention; ACS, acute coronary syndrome; STEMI, ST-elevation myocardial infarction; hs-CRP, high sensitivity C-reactive protein; NT-proBNP, N-terminal pro-brain natriuretic peptide.

### Predictors of MACCE in the very elderly cohort undergoing PCI procedure

**Figure 3:**
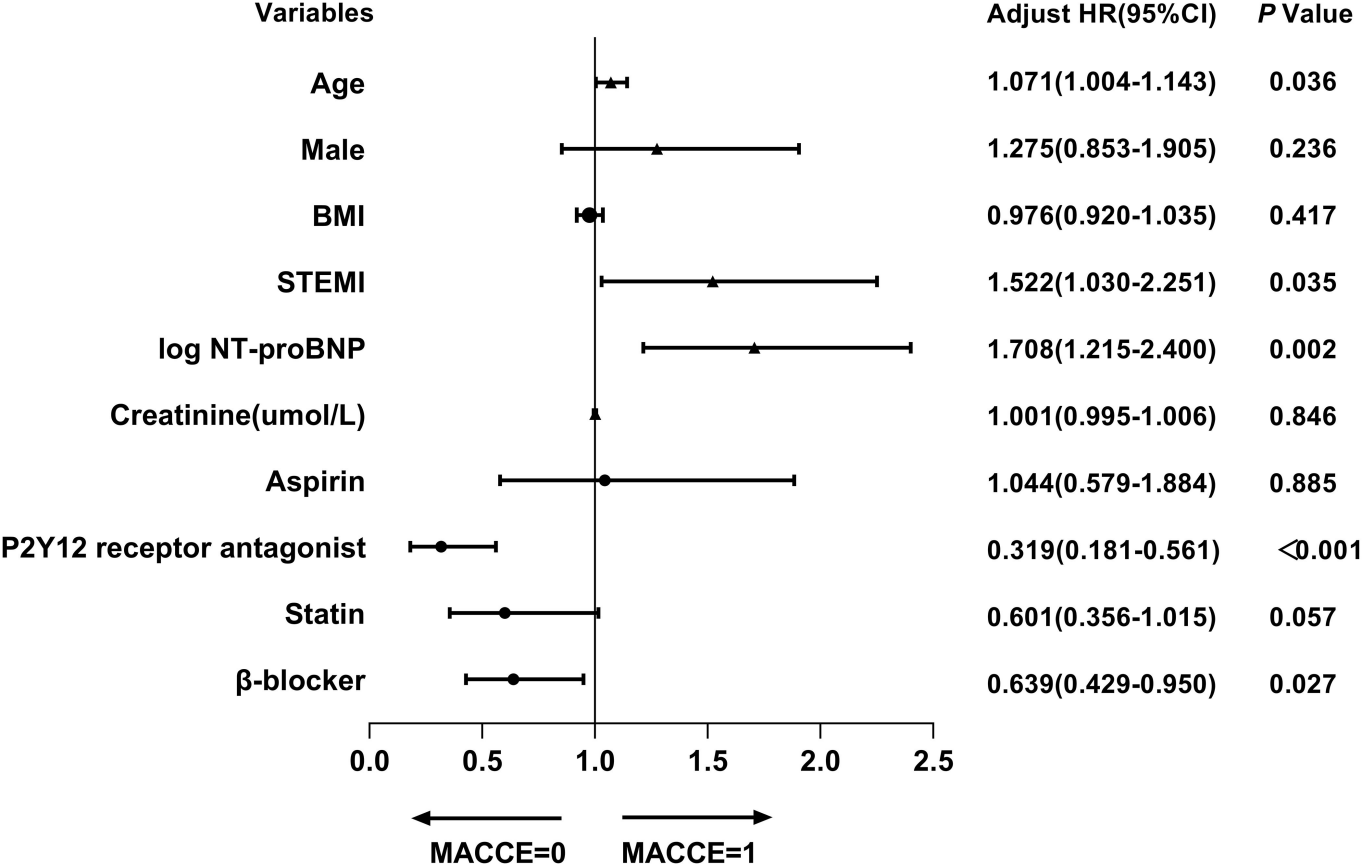
Factors independently associated with composite MACCE in PCI group in multivariable Cox regression analysis. BMI, body mass index; STEMI, ST-elevation myocardial infarction; NT-proBNP, N-terminal pro-brain natriuretic peptide; PCI, percutaneous coronary intervention; MACCE, major adverse cardiovascular and cerebrovascular events.

Univariate Cox regression analysis identified 10 factors, which might be associated with the occurrence of composite MACCE in very elderly patients after PCI, including age, sex, BMI, STEMI, log NT-proBNP, creatinine, and the use of aspirin, P2Y12 receptor antagonist, statin, and β-blocker. Further multivariate analysis confirmed that five factors independently associated with MACCE, involving age, STEMI, log NT-proBNP, and the use of P2Y12 receptor antagonist, and β-blocker. Remarkably, gender was not a related factor to the risk of MACCE in this special population (male: HR 1.275, *p* = 0.236) (Table 4 and Figure 3), which meant that the predictors of MACCE after PCI were similar in both sexes in the present cohort.

**Table 4:** Cox proportional hazards regression analyses for composite MACCE in PCI group.

**Variables**	**Univariate regression**			**Multivariate regression**		
	**HR**	**95% CI**	***p* value**	**HR**	**95% CI**	***p* value**
Age	1.099	1.032–1.171	0.003	1.071	1.004–1.143	0.036
Male	1.205	0.840–1.729	0.311	1.275	0.853–1.905	0.236
BMI	0.944	0.893–0.997	0.040	0.976	0.920–1.035	0.417
STEMI	1.756	1.221–2.527	0.002	1.522	1.030–2.251	0.035
log NT-proBNP	1.936	1.430–2.620	< 0.001	1.708	1.215–2.400	0.002
Creatinine, umol/L	1.005	1.000–1.009	0.056	1.001	0.995–1.006	0.846
Aspirin	0.527	0.325–0.852	0.009	1.044	0.579–1.884	0.885
P2Y12 receptor antagonist	0.358	0.219–0.587	< 0.001	0.319	0.181–0.561	< 0.001
Statin	0.486	0.314–0.751	0.001	0.601	0.356–1.015	0.057
β-blocker	0.597	0.416–0.858	0.005	0.639	0.429–0.950	0.027

p, level of statistical significance.

MACCE, major adverse cardiovascular and cerebrovascular events; PCI, percutaneous coronary intervention; BMI, body mass index; STEMI, ST-elevation myocardial infarction; NT-proBNP, N-terminal pro-brain natriuretic peptide.

### Clinical outcomes in the male and female ACS patients

**Figure 4:**
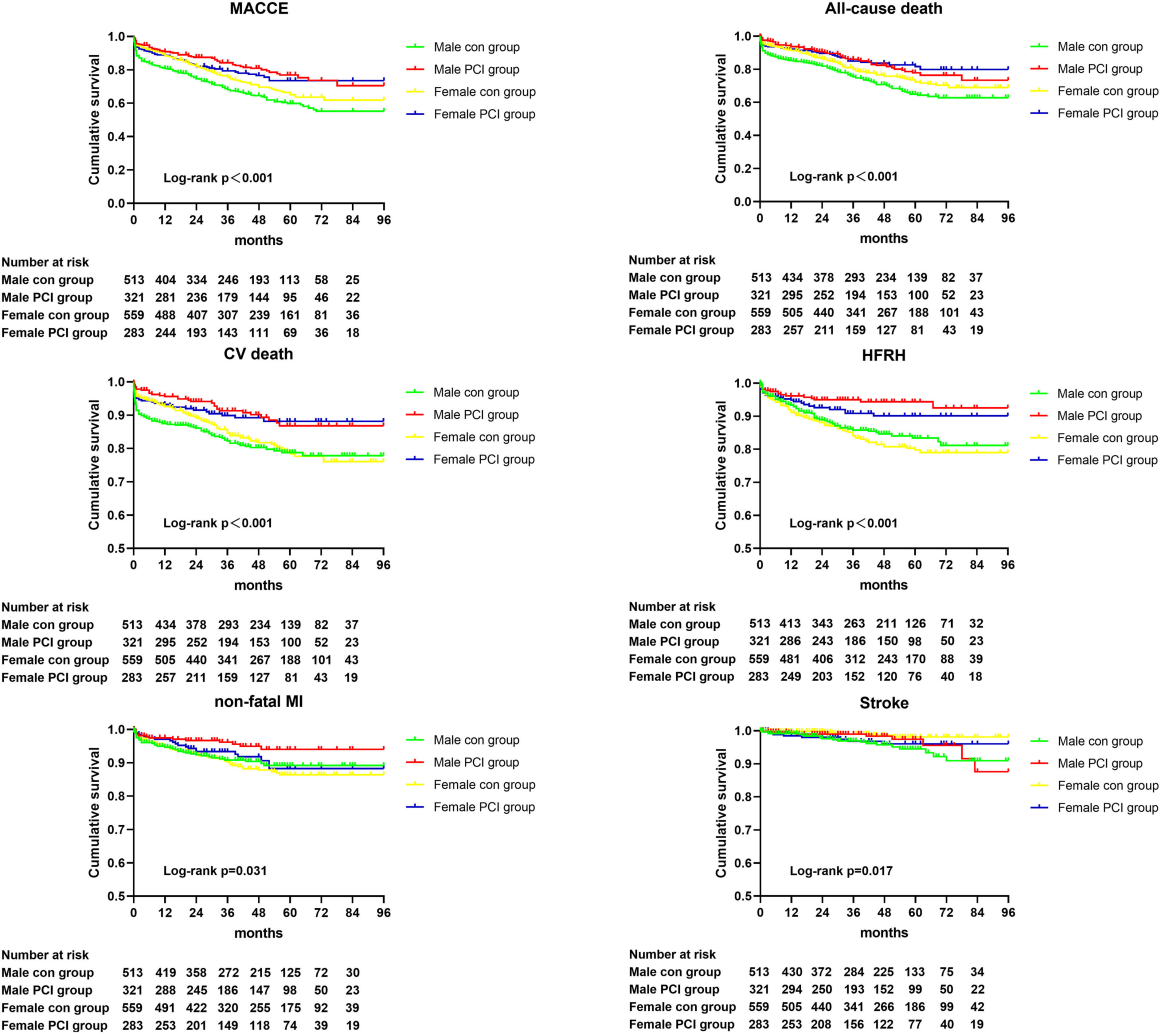
Kaplan–Meier curve analyses for primary and secondary endpoints in PCI and conservative management group by gender. MACCE, major adverse cardiovascular and cerebrovascular events; CV, cardiovascular; HFRH, heart failure requiring hospitalization; MI, myocardial infarction; PCI, percutaneous coronary intervention; con, conservative management.

The primary and secondary endpoints were displayed in Table 5. Compared to conservative management groups, PCI treatment significantly improved primary endpoints (composite MACCE) in both the male (*p* < 0.001) and female (*p* = 0.026) separately in the very elderly ACS population. Furthermore, PCI procedure significantly attenuated the risk of non-fatal MI (*p* = 0.032), HFRH (*p* < 0.001), CV death (*p* < 0.001), and all-cause death (*p* < 0.001) in male ACS patients. In addition, female patients benefited significantly from PCI treatment in the decrease of HFRH (*p* < 0.001), CV death (*p* = 0.005), and all-cause death (*p* = 0.006) when compared to conservative management only. Finally, there were no remarkable differences in the clinical outcomes treated with PCI between sexes during the follow-up period. Kaplan–Meier curves (Figure 4) illustrated and compared the incidences of primary and secondary endpoints of the four groups in detail.

**Table 5:** The comparison of MACCE in PCI and conservative management group by different sexes.

**Variables**	**Male-con**	**Male-PCI**	***p* value**	**Female-con**	**Female-PCI**	***p* value**	***p* value^#^**
	**(*n* = 513)**	**(*n* = 321)**		**(*n* = 559)**	**(*n* = 283)**		
Composite MACCE, n (%)	174 (33.9)	59 (18.4)	< 0.001	156 (27.9)	59 (20.8)	0.026	0.445
Non-fatal MI, n (%)	42 (8.2)	14 (4.4)	0.032	57 (10.2)	21 (7.4)	0.189	0.108
Stroke, n (%)	20 (3.9)	8 (2.5)	0.273	6 (1.1)	8 (2.8)	0.084*	0.798
HFRH, n (%)	65 (12.7)	17 (5.3)	< 0.001	90 (16.1)	23 (8.1)	< 0.001	0.163
CV death, n (%)	94 (18.3)	30 (9.3)	< 0.001	96 (17.2)	28 (9.9)	0.005	0.820
All-cause death, n (%)	148 (28.8)	54 (16.8)	< 0.001	128 (22.9)	42 (14.8)	0.006	0.506
Major bleeding, n (%)	16 (3.1)	10 (3.1)	0.998	4 (0.7)	16 (5.7)	< 0.001	0.125

^*^Fisher's exact test. p, level of statistical significance.

^#^Male-PCI vs. Female-PCI.

MACCE, major adverse cardiovascular and cerebrovascular events; con, conservative management; PCI, percutaneous coronary intervention; MI, myocardial infarction; HFRH, heart failure requiring hospitalization, CV, cardiovascular.

## Discussion

The present study focused on the sex-related differences in PCI treatment in clinical outcomes in very elderly ACS patients. The investigation of differences involved clinical and coronary features, as well as long-term outcomes after PCI and their predictors between the male and female very elderly ACS patients. The results revealed that men tended to have more complex clinical conditions, the male gender was one of the independent factors driving the option of the PCI strategy, and women were less likely to receive PCI procedures in the very elderly cohort. Coronary artery lesions were relatively severe in both sexes, and the ratio of CTO lesions in men was significantly higher than that in women. Furthermore, the PCI procedure significantly decreased the incidence of MACCE in both sexes during follow-up, and there was no gender difference in the benefits of PCI treatment between the sexes. Finally, STEMI, elderly age, and increase in Log NT-proBNP value were independently associated with the risk of MACCE after PCI in the very elderly cohort.

Several studies had shown that there might be some differences between gender of ACS patients in clinical characteristics, efficacy, and safety of PCI procedure, and long-term prognosis (12–14). Very elderly patients are a special group with body hypofunction, more comorbidities, more PCI complications, and high mortality (20). Therefore, very elderly ACS patients have less PCI treatment experience and more concerns. In particular, the sex-related differences in this cohort after PCI is less known. With the aging worldwide, the population of very elderly ACS patients is growing rapidly (20), and the demand for PCI treatment is also increasing. The exploration of efficacy and gender differences of PCI procedure in the very elderly ACS population will contribute to gender-based therapeutic decisions and improve clinical outcomes in this population.

It has been found that female was less likely than male to undergo revascularization in the treatment of ACS in previous studies (9, 21, 22). This study detected the same trend in the very elderly ACS cohort, indicating that the proportion of very elderly female patients receiving PCI was significantly lower than that of the male. Logistic regression analysis verified that male gender was one of the independent factors, driving the option of PCI strategy. Meanwhile, the male tended to be admitted to hospitals with more complex clinical conditions, such as a higher proportion of prior MI, PAD, and smoking, higher levels of hs-CRP, creatinine, and CK-MB, and a lower proportion of LVEF ≥50%. In addition, it was recognized that age, STEMI, level of creatinine, and log NT-proBNP were also driving factors affecting PCI therapeutic decisions.

According to some clinical trials and registries, women had less multivessel disease or left-main disease than men (10, 11), but our study did not find significant differences in three-vessel stenosis and LM disease between genders. The reason may be that the subjects of the present study were a very elderly population, and the proportions of three-vessel stenosis and LM disease were higher in very elderly ACS patients in previous studies (20, 23). However, the CTO ratio in male patients was significantly higher than that in female patients (*p* = 0.007). Despite this, procedural success rates of PCI were relatively high in this very elderly cohort, which was similar to the previous study (24). Importantly, this study found no differences in success rates (*p* = 0.828) and stent numbers (*p* = 0.118) between genders, which demonstrated the feasibility and effectiveness of the PCI procedure in both sexes, especially in females.

PCI procedure had been proven to improve clinical outcomes of elderly patients with ACS (20, 25). The present study demonstrated that PCI treatment synchronously reduced the risk of MACCE in both genders (including composite MACCE, CV death, and HFRH) compared to conservative management. Although the PCI procedure significantly increased the risk of TIMI-major bleeding (*p* < 0.001) in females, it did not offset the benefit of all-cause mortality in women. Meanwhile, this study revealed that the benefit of PCI in improving clinical outcomes was not different between genders. The above results suggested that both male and female very elderly ACS patients should consider more aggressive PCI strategies, especially female patients. Further investigations showed that gender was not the independent factor associated with MACCE after PCI, but age, STEMI, and worse cardiac function independently predicted the risk of MACCE during the follow-up. Also, adherence to P2Y12 receptor antagonist and β-blocker was important, which contributed to decrease the risk of MACCE in the very elderly PCI cohort. Statin was not shown to be associated with the risk of MACCE after PCI during follow-up (HR 0.601, *p* = 0.057) because of the high rates of statin use in this population.

### Limitations

Single-center data and retrospective design are the main limitations of the present study. Treatment strategy reflected the convention and tendency of the single center, which may have an important impact on clinical prognosis. Therefore, the conclusion may have been biased because the objectivity of the results may have been compromised. The retrospective design may miss some characteristics of the patients in the study, such as the evaluation of physical performance and acute kidney injury, which are common in the very elderly cohort (26–28). These factors might have some influences on the clinical outcomes of PCI treatment (26–28). In addition, the small sample size is also a constraint, resulting in an 8-year time span for enrolled patients, and the inability to include more stratified variables.

## Conclusion

Very elderly male ACS patients tended to have more complex clinical conditions and were more likely to receive PCI procedures. PCI treatment had a relatively high procedural success rate and simultaneously improved the long-term clinical outcomes in both male and female very elderly patients.

Besides the descriptions of clinical and coronary differences, the significance of this study is to confirm the benefits of PCI in the very elderly population, especially the benefits without gender differences. So active PCI strategies may be appropriate for very elderly patients, especially females, who should be considered for more aggressive coronary invasive interventions than previously.

## Data availability statement

The original contributions presented in the study are included in the article/supplementary material, further inquiries can be directed to the corresponding author/s.

## Ethics statement

The studies involving human participants were reviewed and approved by Institutional Review Board of Beijing Friendship Hospital. The patients/participants provided their written informed consent to participate in this study.

## Author contributions

J-lW performed the study, statistical analysis, and wrote the manuscript. X-qH contributed to the acquisition of data and analysis and interpretation of data. H-wL and HC provided support and designed the study. C-yG participated in the study data collection. S-mZ contributed to the conception or design and critically revised the manuscript. All authors read and approved the final manuscript.

## Funding

This study was supported by National Key R&D Program of China (2021ZD0111004) and Beijing Key Clinical Subject Program.

## Conflict of interest

The authors declare that the research was conducted in the absence of any commercial or financial relationships that could be construed as a potential conflict of interest.

## Publisher's note

All claims expressed in this article are solely those of the authors and do not necessarily represent those of their affiliated organizations, or those of the publisher, the editors and the reviewers. Any product that may be evaluated in this article, or claim that may be made by its manufacturer, is not guaranteed or endorsed by the publisher.

## Abbreviations

ACS: acute coronary syndrome
PCI: percutaneous coronary intervention
MACCE: major adverse cardiovascular and cerebral events
STEMI: ST-segment elevation myocardial infarction
NT-proBNP: N-terminal pro-brain natriuretic peptide
QoL: quality of life
NSTEMI: non-ST-segment elevation myocardial infarction
UAP: unstable angina pectoris
ECG: electrocardiogram
hs-CRP: high sensitivity C-reactive protein
CK-MB: creatine kinase-MB
ECHO: echocardiography
LVEF: left ventricular ejection fraction
LVEDD: left ventricular end-diastolic diameter
TIMI: thrombolysis in myocardial infarction
MI: myocardial infarction
HFRH: heart failure requiring hospitalization
CV: cardiovascular
HbA1c: Hemoglobin A1c
LM: left main
CTO: chronic total occlusion
HR: hazard ratios
CI: confidence intervals
BMI: body mass index
BP: blood pressures
PAD: peripheral artery disease
TC: total cholesterol
LDL-C: low-density lipoprotein cholesterol
TG: triglyceride
HDL-C: high-density lipoprotein cholesterol
ALT: alanine aminotransferase
IABP: intra-aortic balloon pump
ACEI: angiotensin-converting enzyme inhibitors
ARB: angiotensin receptor blockers.
